# Dissecting the genetic basis of agronomic traits by multi-trait GWAS and genetic networks in maize (*Zea mays* L.)

**DOI:** 10.1007/s42994-025-00241-4

**Published:** 2025-08-14

**Authors:** Ying Zhou, Yanfang Heng, Shoukun Chen, Jinglu Wang, Kunhui He, Jiahui Geng, Kaijian Fan, Yonggui Xiao, Changling Huang, Jiankang Wang, Enying Zhang, Liang Li, Huihui Li

**Affiliations:** 1 College of Agronomy, Qingdao Agricultural University, Qingdao, 266109 China; 2 State Key Laboratory of Crop Gene Resources and Breeding, Institute of Crop Sciences, Chinese Academy of Agricultural Sciences (CAAS), CIMMYT-China Office, Beijing, 100081 China; 3 Nanfan Research Institute, CAAS, Sanya, 572024 China; 4 Beijing Key Lab of Digital Plant, Information Technology Research Center, Beijing Academy of Agriculture and Forestry Sciences, Beijing, 100097 China

**Keywords:** Maize (*Zea mays* L.), Multi-trait, GWAS, Genes, Genetic network

## Abstract

Agronomic traits in maize (*Zea mays* L.) are complex and modulated by pleiotropic loci and interconnected genetic networks. However, the traditional single-trait genome-wide association study (GWAS) method often misses genetic associations among traits, overlooks pleiotropic effects, and underestimates shared regulatory mechanisms. In the current study, we employed multi-trait analysis of GWAS (MTAG) and constructed a genetic network to dissect the genetic architecture of 18 agronomic traits across a genetically diverse panel of 2,448 maize inbred lines. Incorporating MTAG significantly improved the detection of pleiotropic loci that had not been detected by single-trait GWAS. Using a genetic network, we uncovered numerous previously unrecognized connections among traits related to plant architecture, yield, and flowering time. The 49 detected hub nodes, including Zm00001d028840 and Zm00001d033859 (*knotted1*), influence multiple traits. Co-expression analysis of candidate genes across two developmental stages validated their distinct yet complementary roles, with Zm00001d028840 linked to early cell wall remodeling and Zm00001d033849 involved in chromatin remodeling during tasseling. Moreover, we integrated results from GWAS, MTAG, and genetic network analyses to prioritize pleiotropic loci and hub genes that regulate multiple agronomic traits. This integrative approach offers a practical framework for selecting stable, multi-trait-associated targets, thereby supporting more precise and efficient crop improvement strategies.

**Supplementary Information:**

The online version contains supplementary material available at 10.1007/s42994-025-00241-4.

## Introduction

Maize (*Zea mays* L.) is extensively cultivated worldwide (Chen et al. 2017) and is indispensable to global food security. Key agronomic traits directly and indirectly influence maize yield (Ndlovu et al. 2022). Dissecting the genetic basis of these agronomic traits and their underlying regulatory networks is an essential step for developing resilient, high-yielding maize cultivars. Genome-wide association study (GWAS) is a statistical approach that leverages linkage disequilibrium (LD) to identify loci or genes associated with quantitative traits (Hirschhorn and Daly 2005; Korte and Farlow 2013). By analyzing large datasets of molecular markers and their associations with phenotypic traits in diverse populations, GWAS can uncover the genetic basis of these traits (Yan et al. 2010). GWAS has been extensively applied in various plant species (Atwell et al. 2010; Coe et al. 2023; Huang et al. 2010), especially for studying the genetic basis of natural variation and agriculturally important traits (Chu et al. 2024; Li et al. 2013; Lu et al. 2024). In maize, high-density genotyping technologies and rich marker datasets enable the mapping of quantitative traits with GWAS, improving our understanding of the genetic architecture underlying agronomic traits (Sahito et al. 2024).

For the genetic improvement of crops, defining the genetic architecture of agronomic traits represents a highly complex task as such traits can be influenced by multiple major-effect genes, polygenic interactions, epigenetic regulation, and environmental effects (Chen et al. 2024; Delen et al. 2023; Huang et al. 2010). Given the genetic correlations and pleiotropic effects among different traits, single-trait analyses are often insufficient to fully elucidate their underlying mechanisms (Li et al. 2019). By contrast, a joint multi-trait analysis can integrate shared genetic signals, enhance the precision of genetic dissection, and uncover the regulatory networks behind complex traits (Xiong et al. 2022). For example, plant height (PH) and ear height (EH) are critical architectural traits in maize, and they exhibit a strong genetic correlations between them. Both traits are regulated by shared genetic networks and phytohormone signaling pathways (Li et al. 2016; Shu et al. 2023). Genes, such as *Dwarf1* (*ZmDWF1*), *gibberellin 20-oxidase* (*ZmGA20ox*), and *brassinazole-resistant 1* (*ZmBZR1*), regulate both cell elongation and stalk development, thereby coordinately influencing plant architecture and lodging resistance through pleiotropic effects on PH and EH (Cao et al. 2022; Do et al. 2016; Wang et al. 2022). Similarly, days to anthesis (DTA) and days to silking (DTS) are co-regulated by major flowering-time genes (e.g., *CO, CO-LIKE, and TIMING OF CAB* [*ZmCCT*], *Vegetative to generative transition 1* [*Vgt1*], and *pseudo-response regulator 7* [*ZmPRR7*]) that modulate photoperiodic responses under varying environmental conditions, consequently influencing downstream growth and development (Chen et al. 2022; Li et al. 2023; Su et al. 2021). More directly, maize yield requires molecular-, cellular-, and organismal-scale regulations of source-to-sink nutrient translocation. Maintenance of the shoot apical meristem (SAM) by the KNOX family gene *knotted1* (*kn1*) can affect row number per ear (RNPE) (Sentoku et al. 1999; Wu et al. 2025), whereas the downstream target gene of KN1, *growth-regulating factor 4* (*ZmGRF4*), simultaneously modulates kernel weight (KW) and grain size (Zhang et al. 2008).

Genetic networks are complex regulatory systems that are constructed from genes and gene–gene interactions and can help define the genetic basis of biological traits (Han et al. 2023; Lin et al. 2024). The study of genetic networks can deepen our understanding of genetic relationships among different agronomic traits, while guiding strategies for investigating the molecular basis of crop traits (Yang et al. 2022). Recent advances in genomics and bioinformatics analysis, such as genotype–phenotype association analysis, have driven several discoveries related to genetic networks. For example, multi-trait GWAS in soybean (*Glycine max* [L.] Merr.) has highlighted pleiotropic genes that affect both plant architecture and seed traits, and GWAS in wheat (*Triticum aestivum* L.) has revealed complex regulatory networks governing salinity tolerance (Niu et al. 2024; Shan et al. 2022). These findings underscore the potential of network-based approaches, which integrate genetic associations, gene expression data, and functional annotations to reconstruct regulatory relationships, thereby capturing the shared genetic architecture and regulatory mechanisms that underlie complex traits in crops.

In this study, we integrated GWAS, multi-trait analysis of GWAS (MTAG), and genetic network analysis to systematically dissect the genetic architecture of yield-related traits, plant architecture, and flowering time by identifying and characterizing key genes associated with these traits, and by examining their interactions to define their roles and regulatory mechanisms in maize growth and development. Our other objective was to explore the co-expression of several key genes, which would support their co-regulation and potential functional relationships. In addition to describing an integrative approach for exploring the common genetic architecture of multiple traits that influence maize performance, this study also illustrates how knowledge of the genetic basis of maize yield and quality provides theoretical insights that can guide optimization of maize breeding programs.

## Results

### Phenotypic variation and heritability of maize agronomic traits

**Fig. 1:**
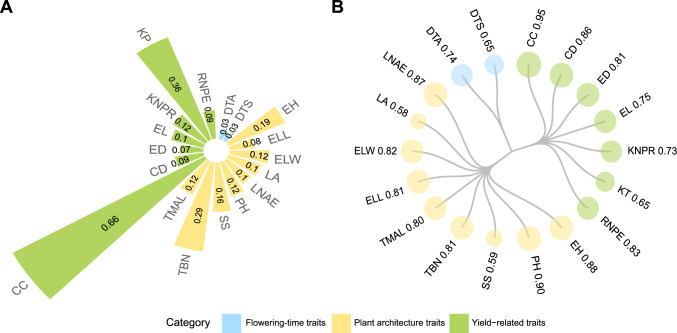
Coefficients of variation and broad-sense heritability of the 18 traits. **A** Coefficients of variation (CVs) for 18 phenotypic traits. CV was calculated as the ratio of the standard deviation to the mean for each trait across three environments, reflecting trait variability. **B** *H*^*2*^ of the 18 traits. Blue, flowering-time traits; yellow, yield-related traits; green, plant architecture traits. Circle sizes are proportional to heritability values. *CC* cob color; *CD* cob diameter; *DTA* days to anthesis; *DTS* days to silking; *ED* ear diameter; *EL* ear length; *ELL* ear leaf length; *ELW* ear leaf width; *EH* ear height; *KNPR* kernel number per row; *KT* kernel type; *LA* leaf angle; *LNAE* leaf number above ear; *PH* plant height; *RNPE* row number per ear; *SS* stalk strength; *TBN* tassel branch number; *TMAL* tassel main axis length

To uncover the genetic architecture underlying 18 agronomic traits predominantly associated with plant architecture and ears in maize, we conducted a descriptive statistical analysis on phenotypes collected from 2,448 inbred lines across three environments in China (Beijing, Henan, Heilongjiang). We observed substantial and continuous phenotypic variation, with coefficients of variation (CVs) ranging from 3 to 66% (Fig. 1A). We classified these 18 traits into three categories based on their functional effects on plant architecture, flowering time, or yield-relatedness (Table S1). Yield-related traits exhibited the highest variability (CV: 7–66%), followed by plant architecture traits (CV: 8–29%), whereas flowering-time traits showed the least variation (CV: 3%). These findings suggest that yield-related and plant architecture traits are likely to be influenced by multiple minor-effect loci with heightened environmental sensitivity, whereas flowering-time traits may be influenced by a few major-effect loci.

To account for the influence of the environment on phenotypic variation, we employed a multi-environment model to reduce noise associated with different environments. The best linear unbiased prediction (BLUP) values across all three environments showed a remarkably lower dispersion than that seen for single-environment observations, indicating that the estimates of genotypic effects are more stable after correcting for environmental influences (Fig. S1). The distributions of phenotypic values for all traits, except for qualitative traits (e.g., cob color and kernel type), followed a quantitative and continuous distribution (Fig. S2). As continuous distributions are characteristic of polygenic traits, these results are consistent with the complexity of the genetic regulation behind each trait analyzed here (Mackay et al. 2009). Broad-sense heritability (*H*^2^) was estimated to range from 0.58 to 0.95 for the 18 agronomic traits (Fig. 1B). *H*^2^ was notably high for PH and EH, with values of 0.88 and 0.90, respectively, indicating that these two plant architecture traits are predominantly controlled by stably inherited genetic factors across generations. By contrast, leaf angle (LA) and stalk strength (SS) had lower heritability (0.58 and 0.59, respectively), suggesting that environmental factors may exert a strong influence on these traits that GWAS might have limited statistical power to dissect such traits. Overall, 14 of the 18 traits had heritability estimates >0.70, thus illustrating the strong influence of genetic factors in determining these traits.

### Phenotypic correlation between maize agronomic traits

**Fig. 2:**
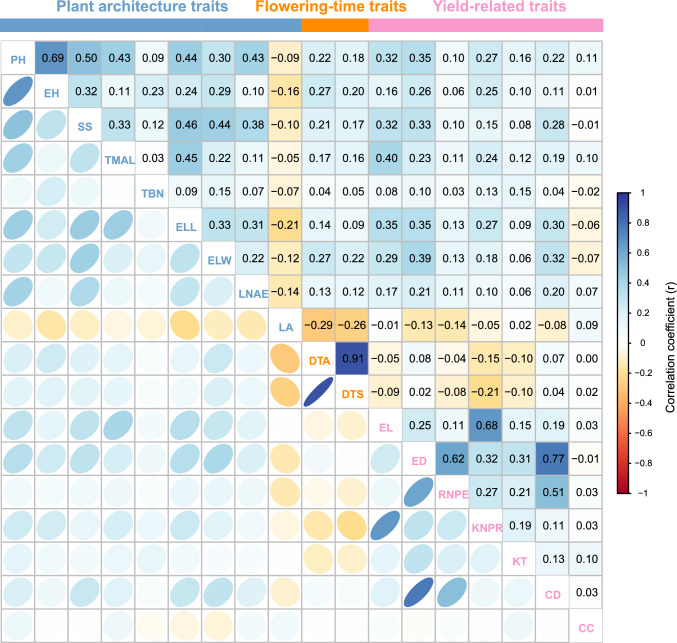
Exploration of pairwise correlations between the 18 traits. Heatmap representation of pairwise correlations between different traits. Pearson correlation coefficients were calculated for each pair of traits. The slope of the ellipse indicates the type of correlation (positive slope indicates positive correlation; negative slope indicates negative correlation). Narrower ellipses indicate stronger correlations, and ellipses that are closer to a circle represent weak or no correlation. Numbers in the upper right triangle represent the Pearson’s correlation coefficients between 1.0 (blue) and −1.0 (red), with the color scale illustrating correlation strength. Text in the diagonal represents the 18 traits. *CC* cob color; *CD* cob diameter; *DTA* days to anthesis; *DTS* days to silking; *ED* ear diameter; *EL* ear length; *ELL* ear leaf length; *ELW* ear leaf width; *EH* ear height; *KNPR* kernel number per row; *KT* kernel type; *LA* leaf angle; *LNAE* leaf number above ear; *PH* plant height; *RNPE* row number per ear; *SS* stalk strength; *TBN* tassel branch number; *TMAL* tassel main axis length

We calculated the Pearson’s correlation coefficients between pairs of traits to illustrate their relationships, using their BLUP values. We detected stronger correlations within traits of the same category than across categories (Fig. 2). For instance, the two plant architecture traits PH and EH showed a significant and positive correlation (*r* = 0.69, *p* < 0.05), possibly because of the shared developmental regulation of internode elongation and stem growth that contributes to both traits. By contrast, inter-category correlations were generally weaker, such as between PH and RNPE (*r* = 0.20, *p* > 0.05), likely because of the differences in regulatory and developmental pathways responsible for these traits. The strongest correlation was between DTA and DTS (*r* = 0.91, *p* < 0.01), highlighting their close biological relationship in the maize reproductive cycle. Pleiotropy and linkage may explain the observed correlations. The observed medium-to-strong correlation coefficients obtained suggest that the 18 traits may share the same underlying genetic or physiological mechanisms and can therefore be used for joint selection to improve breeding efficiency.

### Population structure and genetic diversity within a maize panel of 2,448 inbred lines

**Fig. 3:**
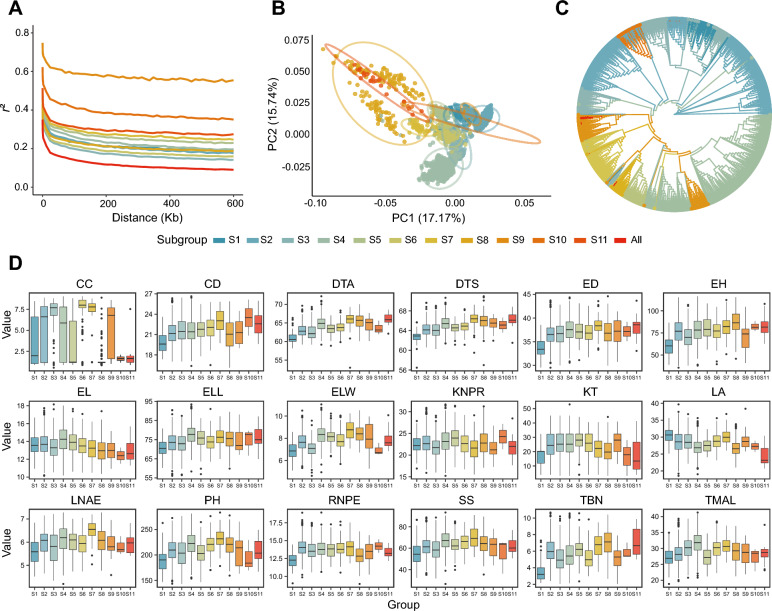
Genetic diversity across the maize panel and its subgroups. **A** Linkage disequilibrium (LD) map for the 11 maize subgroups defined in this study. LD decay was estimated for each of the 11 subgroups based on the squared correlation coefficient values (*r*^2^) plotted against chromosomal distance (kb). **B** Principal component analysis (PCA) plot of the first two eigenvectors for all maize inbred lines, based on genotype information. **C** Neighbor-joining phylogenetic tree of the 2,448 maize accessions. **D** Boxplots of best linear unbiased prediction (BLUP) values for each trait in the 11 subgroups. *CC* cob color; *CD* cob diameter; *DTA* days to anthesis; *DTS* days to silking; *ED* ear diameter; *EL* ear length; *ELL* ear leaf length; *ELW* ear leaf width; *EH* ear height; *KNPR* kernel number per row; *KT* kernel type; *LA* leaf angle; *LNAE* leaf number above ear; *PH* plant height; *RNPE* row number per ear; *SS* stalk strength; *TBN* tassel branch number; *TMAL* tassel main axis length

We performed a semi-supervised ADMIXTURE analysis to evaluate population structure, based on which we classified the 2,448 maize inbred lines into 11 distinct subgroups (S1–S11). We detected higher single-nucleotide polymorphism (SNP) densities at the ends of all chromosomes (Fig. S3). We also observed substantial variation in terms of LD decay patterns across the 11 subgroups (Fig. 3A). Principal component analysis (PCA) using the entire set of genotype data for the 2,448 maize inbred lines revealed that the first two principal components, PC1 and PC2, explain 17.2% and 15.7% of the total genetic variation, respectively (Fig. 3B).

The population structure analysis indicated a substantial overlap in the principal component space of subgroups S8 and S11; S1, S2, and S9; and S3 and S6, suggesting a certain degree of genetic similarity among these subgroups. By contrast, subgroups S4 and S7 were distinctly separated from the other subgroups in the PCA space, reflecting their unique genetic background, adaptive traits, or breeding trajectory. The clustering pattern observed in the phylogenetic tree largely aligned with that obtained with the PCA, confirming the genetic similarities and differences among specific subgroups (Fig. 3C). When we plotted the BLUP values for each phenotypic trait across subgroups, we noticed that subgroups S4 and S7 reached higher values than the other subgroups for PH, ear leaf length (ELL), and ear leaf width (ELW) (Fig. 3D). The BLUP values for multiple traits, including DTA and DTS, were highly similar for subgroups S8 and S11, suggesting a possible common genetic background or similar artificial selection pressures during their breeding histories.

### Identification of loci by GWAS and their functional analysis

**Fig. 4:**
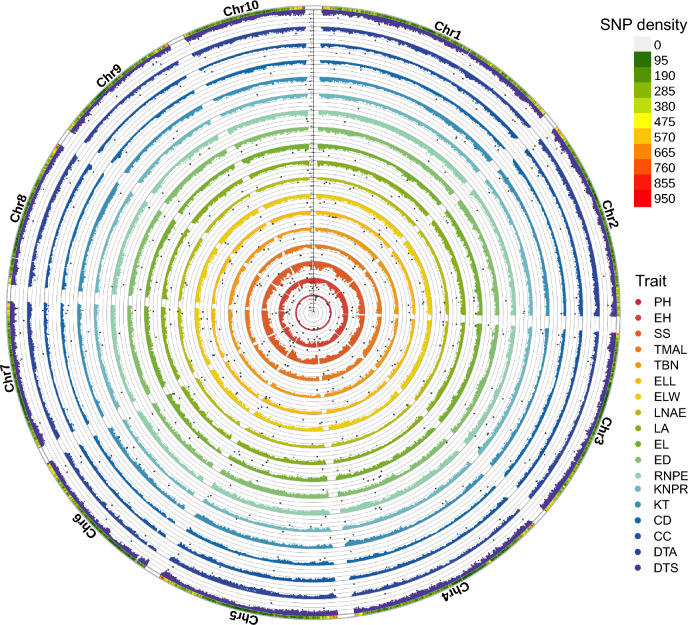
GWAS of the 18 traits based on the FarmCPU model. Circular Manhattan plot of association results for the 18 traits across the maize genome. Chromosomes (Chr 1–Chr 10) are arranged in adjacent segments. The genomic positions of significant SNPs were plotted on the circles representing each trait. The outer ring shows SNP density according to the color gradient in the upper right corner. *CC* cob color; *CD* cob diameter; *DTA* days to anthesis; *DTS* days to silking; *ED* ear diameter; *EL* ear length; *ELL* ear leaf length; *ELW* ear leaf width; *EH* ear height; *KNPR* kernel number per row; *KT* kernel type; *LA* leaf angle; *LNAE* leaf number above ear; *PH* plant height; *RNPE* row number per ear; *SS* stalk strength; *TBN* tassel branch number; *TMAL* tassel main axis length

To identify the genetic loci underlying these agronomic traits, we conducted GWAS using the Fixed and Random Model Circulating Probability Unification (FarmCPU) model, incorporating the 11 principal components derived from a PCA of the genotype data as covariates to account for population structure. This analysis yielded a total of 558 significant SNPs for the 18 traits based on single-trait GWAS results (Fig. 4; Fig. S4). We identified 457 candidate genes located within the regions 10 kb upstream or downstream of significant SNPs, of which 368 have functional annotations (Table 1). To explore the possible biological functions of these candidate genes, we conducted a gene ontology (GO) functional enrichment analysis. This analysis revealed significant enrichment in ‘polyol transmembrane transporter activity’, ‘organic hydroxy compound transmembrane transporter activity’, and ‘alcohol transmembrane transporter activity’ (Table S2). These activities are localized to the plasma membrane and cell periphery, suggesting roles in transmembrane solute transport and osmotic regulation, which could function coordinately to balance stress acclimation with metabolic homeostasis.

**Table 1:** Summary of GWAS results for the 18 traits analyzed in this study

Trait	SNPs	Genes	Mean PVE (%)	Min PVE (%)	Median PVE (%)	Max PVE (%)
CC	52	35	1.89	0.89	1.46	17.20
CD	30	26	1.23	0.89	1.18	2.10
DTA	25	21	1.21	0.87	1.26	1.50
DTS	24	19	1.58	0.95	1.65	2.97
ED	29	18	1.40	0.87	1.16	3.44
EH	43	33	1.35	0.85	1.20	4.06
EL	36	30	1.25	0.86	1.13	2.36
ELL	31	27	1.32	0.86	1.11	4.21
ELW	26	17	1.36	0.90	1.20	2.43
KNPR	35	28	1.24	0.88	1.15	2.31
KT	23	20	1.16	0.86	1.09	1.96
LA	24	22	1.27	0.90	1.21	2.13
LNAE	31	24	1.60	0.87	1.33	3.04
PH	36	30	1.40	0.86	1.24	2.83
RNPE	27	25	1.42	0.87	1.27	3.48
SS	24	19	1.35	0.86	1.33	2.86
TBN	28	24	1.35	0.86	1.25	2.29
TMAL	34	25	1.25	0.86	1.24	2.16

Note: Mean percentage of variance explained (PVE), Min PVE, Median PVE, Max PVE, are based on the significant SNPs only. *CC* cob color; *CD* cob diameter; *DTA* days to anthesis; *DTS* days to silking; *ED* ear diameter; *EL* ear length; *ELL* ear leaf length; *ELW* ear leaf width; *EH* ear height; *KNPR* kernel number per row; *KT* kernel type; *LA* leaf angle; *LNAE* leaf number above ear; *PH* plant height; *RNPE* row number per ear; *SS* stalk strength; *TBN* tassel branch number; *TMAL* tassel main axis length

When we compared these candidate genes with previously reported genes and loci mapped in earlier GWAS and by quantitative trait locus (QTL) studies, 27% (101/457) of the candidate genes from GWAS had previously been identified in studies of agronomic traits, such as yield (Shen et al. 2022), quality (Qu et al. 2024), plant height (Gao et al. 2024), or stress resistance (Xiao et al. 2024) (Table S3). Notably, we identified four candidate genes as being significantly associated with more than one trait, suggesting that these genes might have pleiotropic effects or shared regulatory pathways. Besides validating some previously identified loci, these results uncovered potential co-regulatory mechanisms among multiple traits.

### Detection of pleiotropic loci by MTAG

**Fig. 5:**
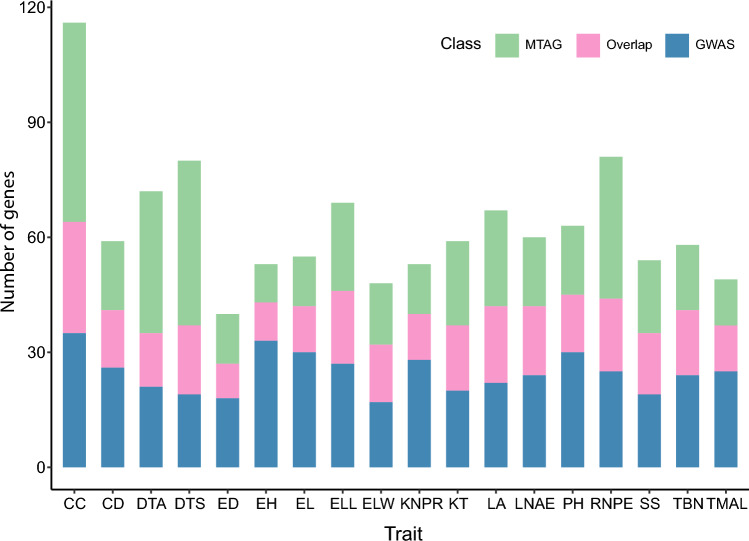
Number of candidate genes detected for each trait by MTAG, GWAS, or both methods. Stacked bar plot showing the number of significant candidate genes associated with each of the 18 traits identified through GWAS, MTAG, or both analyses. Traits are shown on the *x*-axis; gene counts are shown on the *y*-axis. Blue, genes identified only by GWAS; green, genes identified only by MTAG; pink, genes detected in both analyses. *CC* cob color; *CD* cob diameter; *DTA* days to anthesis; *DTS* days to silking; *ED* ear diameter; *EL* ear length; *ELL* ear leaf length; *ELW* ear leaf width; *EH* ear height; *KNPR* kernel number per row; *KT* kernel type; *LA* leaf angle; *LNAE* leaf number above ear; *PH* plant height; *RNPE* row number per ear; *SS* stalk strength; *TBN* tassel branch number; *TMAL* tassel main axis length

Pleiotropy is the situation in which a single gene or locus influences multiple traits (Mackay and Anholt 2024). Identification of pleiotropic loci is important for understanding the genetic connections between traits, with MTAG improving this search by allowing simultaneous analysis of multiple traits to detect shared genetic mechanisms. Analysis by MTAG identified 546 significant SNPs located near 435 candidate genes (Fig. 5; Table S4). MTAG and GWAS had a total of 361 significant SNPs in common, indicating strong statistical support for these SNPs across different analytical methods. Furthermore, MTAG uncovered 182 significant loci not previously reported to be associated with any trait in single-trait GWAS, suggesting that the enhanced statistical power of MTAG enables the detection of complex genetic architectures that are masked in a single-trait analysis. Notably, MTAG detected several more potentially pleiotropic loci, including 17 SNPs associated with at least two traits. Fourteen of these pleiotropic loci were associated with DTA and DTS, two highly correlated traits that influence maize reproductive development (Buckler et al. 2009). Moreover, MTAG uncovered several pleiotropic loci associated with multiple traits not previously detected by single-trait GWAS owing to their moderate effect sizes for individual traits. This improvement may stem from the ability of MTAG to integrate genetic correlations across traits, enhancing statistical power to identify complex regulatory hubs.

### Construction of the genetic network for multiple agronomic traits

**Fig. 6:**
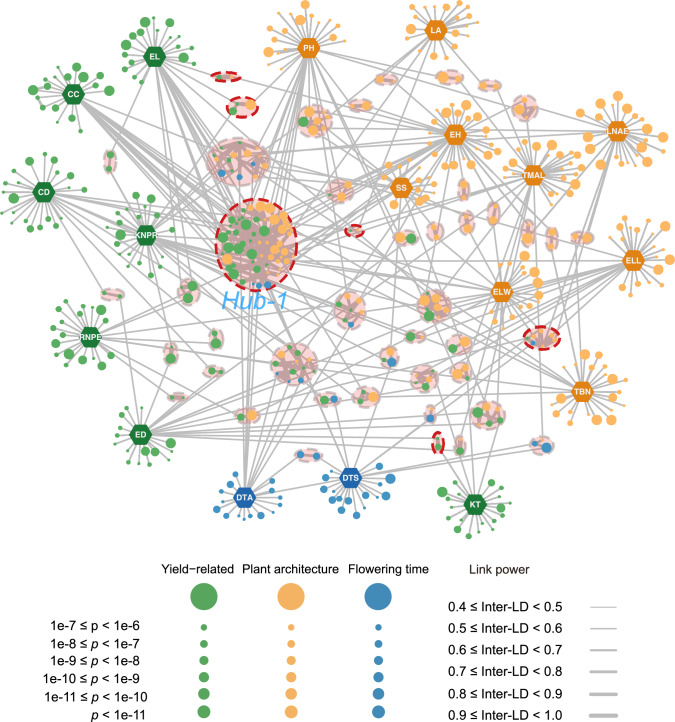
Association network of the significant peaks identified by GWAS for the 18 maize traits. The genetic network was constructed for loci detected by GWAS. Hexagons, agronomic traits; filled circles, significant peaks; open ellipses, hubs (*n* = 49); red dashed ellipses, significant genomic regions containing known genes. The nodes represent 18 traits and their respective significantly associated genomic regions. The edges between the genomic regions of different traits were linked by Inter-LD. Only edges with an average LD ≥ 0.4 are displayed. *CC* cob color; *CD* cob diameter; *DTA* days to anthesis; *DTS* days to silking; *ED* ear diameter; *EL* ear length; *ELL* ear leaf length; *ELW* ear leaf width; *EH* ear height; *KNPR* kernel number per row; *KT* kernel type; *LA* leaf angle; *LNAE* leaf number above ear; *PH* plant height; *RNPE* row number per ear; *SS* stalk strength; *TBN* tassel branch number; *TMAL* tassel main axis length

To explore the possible interrelatedness among the 18 agronomic traits in maize and identify the genetic regulatory mechanisms underlying these complex traits, we constructed separate genetic networks based on the GWAS and MTAG results. The GWAS-derived network comprised 531 significantly associated genomic regions, with 327 regions linked to single traits and 204 regions connected through inter-linkage disequilibrium (Inter-LD) associations (Fig. 6). Among these regions, 31 were associated with two traits, and 18 served as “central hubs” (a subset of highly connected hub regions linked to three or more traits), suggesting their pivotal regulatory roles across multiple traits (Table S5). The genetic network derived from the MTAG results exhibited stronger connectivity than the network based on the single traits identified by GWAS, consisting of 141 regions associated with single traits and 310 Inter-LD associations (Fig. S5). A comparative analysis of the two networks revealed 252 shared significant genomic regions, several of which overlapped with known regulatory genes, reinforcing their potential functional significance.

To further elucidate the regulatory roles of the 49 hub regions in maize agronomic traits that were identified based on the single-trait GWAS-derived genetic network, we examined the genes located within these hubs and identified several key candidates involved in growth, development, and stress responses. In particular, hub-1, serving as the largest central hub, and encompassing several genomic regions, was associated with various maize traits. For example, the QTL411 region, associated with PH, contained several well-known genes, such as Zm00001d033850 (*tubulin a1* [*tua1*]), Zm00001d033859 (*kn1*), and Zm00001d033861 (*knotted-related homeobox3* [*kno3*]), which participate in microtubule formation and regulation of cell division (Dong et al. 2023; Guo et al. 2022; Kerstetter et al. 1997; Skibbe et al. 2009). Additionally, genomic regions engaged in high Inter-LD with QTL411, such as QTL450 and QTL80, contained Zm00001d032794 (*xyloglucan galactosyltransferase 1* [*xgat1*]) and the well-known dwarfing gene Zm00001d031926 (*ZmGA20ox1*), respectively. Other notable regions within hub-1 included QTL1, which contained Zm00001d028826 (*root hair defective 3* [*rth3*]), a gene regulating both root and aboveground traits (Liu et al. 2023). In the QTL342 region, Zm00001d033572 (*auxin-regulated protein 1* [*auxrp1*]) was previously associated with resistance to maize stalk rot, suggesting a likely function in plant disease resistance (Hou et al. 2021). Similarly, QTL206 harbored the Zm00001d028185 (*remorin 1* [*remo1*]) gene, which was previously linked to disease resistance as well (Zhang et al. 2020). Additionally, Zm00001d028759 (*pyruvate decarboxylase 3* [*pdc3*]) was close to QTL23 and was shown to regulate sugar metabolism and energy allocation, therefore influencing growth and stress tolerance in maize (Stagnati et al. 2020).

These detected connections among traits related to plant architecture, yield, and flowering time in our genetic network may reflect shared regulatory mechanisms. These findings provide a comprehensive framework for investigating the molecular interactions between the genes involved, and offer new targets for functional validation and breeding applications.

### Gene co-expression analysis in maize

**Fig. 7:**
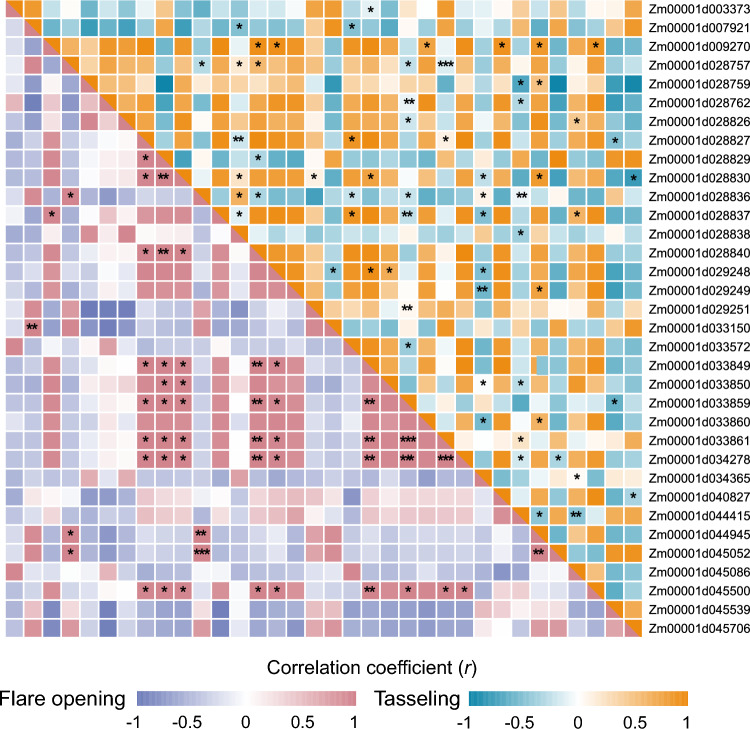
Heatmap of gene co-expression dynamics in maize. Heatmap representation of correlation in expression levels at the flare opening (lower left) or tasseling (upper right) stage. The color scales represent positive (red or orange) or negative (blue or purple) correlations. The right *y*-axis lists gene ID numbers. Significant correlations were determined at the level of *P < 0.05, ** *P* < 0.01, or ***P < 0.001

To verify the synergistic interactions between the candidate genes detected in the genetic network inferred from both GWAS and MTAG analyses, we conducted reverse-transcription quantitative PCR (RT-qPCR) to assess the expression levels of several candidate genes across 12 maize tissues from the B73 inbred line, collected at the flare opening (V12) and tasseling (VT) stages (Table S6). We then calculated the Pearson’s correlation coefficient for expression between gene pairs to assess the relationships in their expression patterns (Fig. 7). Among the 30 selected gene pairs, nine pairs exhibited significant co-expression at the flare opening stage, and seven pairs were co-expressed at the tasseling stage, thus supporting their involvement in the same genetic network or pathways (Table 2). In the QTL411 region, genes, such as Zm00001d033859 (*kn1*), Zm00001d033861 (*kno3*), and Zm00001d033850 (*tua1*), were all co-expressed with one another as well as with other genes at different developmental stages. Notably, the gene pairs Zm00001d029248 and Zm00001d033849, Zm00001d028840 and Zm00001d033849, and Zm00001d028840 and Zm00001d034278 showed significant correlations in their expression levels at both stages. These results validated the genetic network inferred from genomic analysis and the potential synergistic roles of genes in similar biological functions, and further revealed the dynamics of gene expression across different growth stages of maize, deepening our understanding of the genetic regulation underlying maize yield, plant architecture, and flowering-time traits.

**Table 2:** Significantly associated gene pairs at the flare opening and tasseling stages

Gene 1	Gene 1 location (Chr: bp)	Gene 2	Gene 2 location (Chr: bp)	Pearson's correlation coefficient	FDR	Stage
Zm00001d029248	1:63,708,680–63,711,620	Zm00001d033849	1:275,752,027–275,757,102	0.988	0.025	Flare opening
Zm00001d028840	1:48,333,284–48,337,489	Zm00001d033849	1:275,752,027–275,757,102	0.997	0.008	Flare opening
Zm00001d028830	1:47,982,359–47,988,628	Zm00001d033850	1:275,839,810–275,847,427	0.988	0.026	Flare opening
Zm00001d028840	1:48,333,284–48,337,489	Zm00001d033859	1:276,073,335–276,081,242	0.999	0.004	Flare opening
Zm00001d028830	1:47,982,359–47,988,628	Zm00001d033859	1:276,073,335–276,081,242	0.991	0.021	Flare opening
Zm00001d028829	1:47,880,745–47,883,443	Zm00001d033861	1:276,344,334–276,366,296	0.994	0.014	Flare opening
Zm00001d028829	1:47,880,745–47,883,443	Zm00001d034278	1:288,354,743–288,358,020	0.994	0.014	Flare opening
Zm00001d028840	1:48,333,284–48,337,489	Zm00001d034278	1:288,354,743–288,358,020	0.999	0.003	Flare opening
Zm00001d045052	9:11,181,061–11,182,524	Zm00001d045086	9:12,074,059–12,079,631	1.000	0.000	Flare opening
Zm00001d028840	1:48,333,284–48,337,489	Zm00001d033572	1:267,360,211–267,364,567	0.945	0.027	Tasseling
Zm00001d029249	1:63,744,994–63,752,712	Zm00001d033849	1:275,752,027–275,757,102	0.912	0.045	Tasseling
Zm00001d028837	1:48,282,040–48,282,929	Zm00001d033849	1:275,752,027–275,757,102	0.978	0.009	Tasseling
Zm00001d029248	1:63,708,680–63,711,620	Zm00001d033849	1:275,752,027–275,757,102	0.970	0.012	Tasseling
Zm00001d028840	1:48,333,284–48,337,489	Zm00001d033849	1:275,752,027–275,757,102	0.957	0.023	Tasseling
Zm00001d028837	1:48,282,040–48,282,929	Zm00001d034278	1:288,354,743–288,358,020	0.939	0.028	Tasseling
Zm00001d028840	1:48,333,284–48,337,489	Zm00001d034278	1:288,354,743–288,358,020	0.994	0.002	Tasseling

*FDR* false discovery rate

## Discussion

This study aimed to uncover the genetic basis of 18 agronomic traits in maize using GWAS and MTAG. We constructed a genetic network to explore relationships among traits and identified loci potentially involved in regulating multiple traits. These analyses were conducted in a large maize population of 2,448 inbred lines to provide sufficient statistical power to ensure robust results.

### Dual roles of inbred lines in maize genetics and hybrid improvement

The inbred lines used in this study exhibit extensive genetic diversity, encompassing key heterotic groups and are widely used as breeding germplasm for modern maize improvement. The panel includes Iowa Stiff Stalk Synthetic lines, Lancaster Surecrop lines, and elite Chinese inbred lines, each representing distinct genetic backgrounds that serve as foundational parental lines in commercial hybrid development (Bornowski et al. 2021; Wang et al. 2023). The inclusion of both temperate and subtropical germplasm enhanced the adaptability potential of the panel across diverse environments, and the presence of historically significant inbred lines ensured continuity in breeding progress through their continued use as parental lines across breeding generations. Population structure analysis and PCA confirmed that these lines form genetically distinct clusters, aligning with known heterotic groups and breeding lineages.

Hybrid breeding remains the ultimate goal in maize improvement because hybrids benefit from heterosis, resulting in superior yield, stress resilience, and broad adaptability, but inbred lines remain indispensable for genetic research (Cui et al. 2020; Fu et al. 2022). Their stable genetic background and faster LD decay compared to hybrid populations and landraces enable precise mapping of trait-associated loci, facilitating the discovery of key genetic variants (Wang et al. 2020). Additionally, their genetic uniformity (characterized by a high level of homozygosity and minimal heterozygosity) allows for controlled experiments and reproducible results. However, inbred lines also have inherent limitations: they lack heterosis, often exhibiting lower vigor and yield than their hybrids, and may not fully capture epistatic interactions and gene expression dynamics found in commercial hybrids (Hochholdinger and Yu 2025). Nevertheless, by leveraging inbred lines for genetic dissection and validating key findings in hybrid populations, this study contributes to marker-assisted selection and genomic selection, ultimately accelerating maize hybrid improvement.

### Advantages of integrating GWAS and MTAG

GWAS demonstrates a robust capacity for identifying genetic loci for individual traits. In this work, we identified 558 SNPs across 457 genes significantly associated with 18 agronomic traits by GWAS. Many of these genes have been previously characterized and modulate traits related to maize growth, development, stress resistance, and yield. For example, Zm00001d039283 (*glutamate-like receptor 1* [*ZmGLR1*]), a candidate gene for ELL, encodes a cell membrane–localized microtubule-associated protein affecting leaf morphogenesis (Wang et al. 2019). By regulating microtubule dynamics, *ZmGLR1* may also influence cell elongation and division, thereby directly affecting ear length. Similarly, deletion of Zm00001d039634 (*ZmGA3ox2*), a candidate gene for SS that encodes a GA_3_ β-hydroxylase involved in gibberellin biosynthesis, leads to disrupted GA biosynthesis, resulting in dwarfism and lodging resistance (Teng et al. 2013). Additionally, Zm00001d034212 (*knox8*), a candidate gene for EH, belongs to the *KNOX* family and is specifically expressed in the SAM and young stems (Bharathan et al. 1999). Its expression pattern is similar to that of Zm00001d033859 (*kn1*), suggesting that it may influence stem internode elongation, stem thickness regulation, and meristem differentiation through similar mechanisms (Luo et al. 2024). As a result, *knox8* may affect important agronomic traits, such as PH and EH.

MTAG integrates GWAS summary statistics from multiple traits and significantly improves the identification of pleiotropic genes and shared genetic mechanisms across traits by considering their genetic correlations. The advantage of this method is particularly evident in highly correlated traits, such as DTA and DTS. In this study, MTAG uncovered several pleiotropic loci that were not previously detected through traditional GWAS. Among these loci, we identified several candidate genes with potential functional significance. For example, MTAG identified the subtilisin family gene Zm00001d017522 (*subtilisin 19* [*sbt19*]), which was associated with both DTA and DTS, and is known to participate in protein processing, cell wall remodeling, and plant reproductive development, suggesting its potential involvement in flowering-time regulation (Hou et al. 2023). MTAG also indicated that the Amino Acid/Auxin Permease family member Zm00001d017557 (*AAAP35*) is a key gene involved in multiple agronomic traits, likely contributing to amino acid transport and phytohormone signaling crucial for plant growth and stress acclimation (Pankievicz et al. 2022). Additionally, the basic helix–loop–helix (bHLH) transcription factor gene Zm00001d017559 (*ZmbHLH127*), which may regulate phytohormone-dependent development, may influence flowering time and yield-related traits (Thoben and Pucker 2023). Importantly, the effectiveness of MTAG is influenced by the strength of genetic correlations between traits. When genetic correlations are weak, this method may fail to capture cross-trait signals, potentially leading to lower detection power (Xu et al. 2023).

In conclusion, our findings underscore the complementary strengths of GWAS and MTAG in dissecting the genetic architecture of complex agronomic traits, and results obtained by this integrated approach provide a foundation for prioritizing candidate genes for subsequent functional validation to improve maize productivity, resilience, and adaptability. However, for MTAG to better capture cross-trait genetic signals from weakly correlated traits, further modification and optimization are needed to improve its performance.

### Genetic regulatory mechanisms revealed by genomic association networks

LD reflects genetic associations between different genes or SNPs within a given genome, and the genetic background of phenotypic traits is often evoked through these association loci (Slatkin 2008). In this study, GWAS identified 558 significant SNPs associated with 18 traits, enabling the construction of genetic networks based on Inter-LD values among these SNPs. The reconstructed genetic network revealed genetic relatedness among traits and showed that loci can act synergistically to jointly influence complex phenotypes in maize.

Genetic networks are of substantial scientific and practical significance and provide an extensive conceptual framework for understanding the mechanisms underlying genetic associations among traits, thus facilitating prioritization of genomic regions linked to multiple traits based on their network relationships. For instance, the identification of pleiotropic loci allows breeders to optimize multiple agronomic traits in a single breeding cycle, which can accelerate the development of high-yielding, stress-resilient varieties. In addition, close examination of hub nodes can help identify genes with a central role in regulating multiple traits, offering potential targets for enhancing maize performance in breeding programs. For example, *kn1* is a key regulatory gene that maintains meristematic activity and regulates the cell cycle. As a central node in hub-1 of our genetic network, *kn1* interacted significantly with Zm00001d032794 (*xgat1*) and Zm00001d031926 (*ZmGA20ox1*), suggesting their close involvement in regulating maize plant growth and development. XGAT1, a crucial enzyme in cell wall glycosylation, participates in cell wall biosynthesis and remodeling, influencing cell wall mechanical strength and cell expansion, both of which are critical for plant growth (Kozlova et al. 2020). ZmGA20ox1 is a gibberellin 20-oxidase that regulates gibberellin biosynthesis and plant growth (Song et al. 2011). The interactions between *kn1* and these genes highlight its central role in the genetic regulatory network of maize, coordinating pathways, such as cell division, phytohormone biosynthesis, and cell wall modification, to regulate multiple agronomic traits.

Prioritizing hub genes such as *kn1* can provide valuable insights for functional validation studies and molecular breeding programs aimed at optimizing plant architecture and enhancing yield stability. Overall, genetic networks can help expand our perspective of complex trait regulation and facilitate the optimization of crop breeding strategies.

### Synergistic roles of co-expressed genes in maize growth and development

Gene co-expression signifies the coordinated regulation of genes that participate in similar pathways, including metabolic pathways, signal transduction, and stress responses, ultimately providing insight into the complex mechanisms for regulating maize acclimation to environmental changes and developmental signals (Bertolini et al. 2025; Tian et al. 2018).

Analysis of gene co-expression patterns revealed that the expression profile of Zm00001d028840 is significantly positively correlated with several other genes at the flare opening stage. This gene encodes a protein containing a domain of unknown function 4378 (DUF4378) and shares 72% sequence similarity with the C-terminal region of Grain width 7 (*OsGW7*), which regulates cell wall composition through interactions with xyloglucan endo-transglycosylases in rice (*Oryza sativa*) (Ma et al. 2017). Based on its sequence similarity and functional annotation, the protein encoded by Zm00001d028840 is likely involved in cell wall remodeling during early development, potentially modulating cell expansion and organogenesis. By contrast, Zm00001d033849 expression levels were significantly positively correlated with those of various genes at the maturation stage. As a component of the COMPASS-like H3K4 histone methylase complex, WDR5A is likely involved in chromatin remodeling and transcriptional regulation, critical for modulating gene expression at later developmental stages. For example, Zm00001d033849 may regulate the expression of stress-responsive genes or yield-related pathways, consequently affecting maize acclimation potential to environmental challenges and reproductive success. The dynamic co-regulation of these genes across different growth phases indicates that they may exhibit distinct yet potentially complementary roles in maize development.

The synergistic interactions between co-expressed genes highlight the complexity of genetic regulation in maize, requiring further research and confirmation. Further functional validation, such as through gene editing or transcriptomic profiling under stress conditions, may provide crucial evidence of their specific contributions to developmental transitions, stress tolerance, and agronomic performance, paving the way for targeted breeding of maize lines with enhanced productivity and resilience.

## Conclusion

In summary, this study leverages GWAS to dissect the genetic basis of 18 key agronomic traits in maize, identifying 558 significant SNPs located at or near 457 functionally annotated genes. By integrating GWAS with MTAG, we uncovered pleiotropic loci sharing genetic mechanisms and revealed intricate connections among traits related to plant architecture, yield, and flowering time. Notably, the reconstruction of a comprehensive genetic network highlighted critical hub nodes, such as Zm00001d033859 (*kn1*) and Zm00001d028840, which play central roles in coordinating multiple traits. This network elucidates the genetic interdependence of agronomic traits and provides a strategic framework for prioritizing significant genomic regions linked to multiple traits in breeding programs. Future research should integrate omics data and refine tools such as MTAG to enhance the detection of cross-trait signals, particularly for traits with weak genetic correlations. By constructing and analyzing genetic networks, our study provides a solid foundation for precision breeding aimed at optimizing trait coordination and addressing global agricultural challenges.

## Materials and methods

### Plant materials and phenotyping analysis

The experimental material for this study was a genetically diverse panel comprising 2,448 commercial maize (*Zea mays* L.) inbred lines. The panel comprises historically significant and recently improved inbred lines, as well as breeding and research materials contributed by multiple institutions and agricultural biotechnology companies (Fan et al. 2025). All inbred lines were planted and phenotyped at three experimental sites in China in 2021: Harbin, Heilongjiang (45.66°N, 126.61°E); Xinxiang, Henan (35.19°N, 113.80°E); and Shunyi District, Beijing (39.91°N, 116.39°E). To account for multiple test corrections, analysis of variance (ANOVA) was used to assess the significance of differences in traits across environments. In addition, relationships between traits were assessed by calculating their pairwise Pearson’s correlation coefficients. Descriptive statistical analysis, ANOVA, and correlation analysis were carried out using phenotypic data as input; the corresponding graphs were plotted using R software (version 4.3.3). Phenotypic data were preprocessed to identify and remove outliers, defined as values falling outside the 1.5 × interquartile range (IQR × 1.5), which were considered potential measurement errors. To eliminate the influence of environmental effects and other non-genetic factors on phenotypic values, a mixed linear model was used to estimate best linear unbiased prediction (BLUP) values for each of the 18 traits using the ‘lme4’ R package for subsequent application in GWAS (Bates et al. 2015). *H*^2^ was calculated using the following formula:

$$\begin{array}{c}{H}^{2}=\frac{{V}_{g}}{{V}_{g}+\frac{{V}_{ge}}{L}+\frac{{V}_{e}}{RL}}\end{array}(1)$$

where ${V}_{g}$ is the genetic variance, ${V}_{ge}$ is the variance for genotype-by-environmental interactions, ${V}_{e}$ represents the error variance, $L$ is the number of sites, and $R$ is the number of replications.

### DNA extraction, sequencing, and data processing

Genomic DNA was extracted from young maize leaf tissue using a GenoPrep DNA Rapid Extraction Kit (Magnetic Beads, MOLBREEDING, Shijiazhuang, China). Resequencing of the 2,448 maize inbred lines was performed on a MGISEQ-T7 instrument to a sequencing depth of 1× . Quality control was performed using PLINK (version 1.9) (Purcell et al. 2007), and single-nucleotide polymorphisms (SNPs) with a missing rate above 20% and a minor allele frequency below 5% were excluded from the genotyping dataset, resulting in a set of 436,944 high-quality SNPs for use in analyses.

### Population structure analysis

LD analysis was performed for each subpopulation using PLINK (version 1.9) to calculate *r*^2^ for any two SNPs on a chromosome. LD decay maps were plotted using mean *r*^2^ values for distances of up to 600 kb using PopLDdecay (Zhang et al. 2019) software. In addition, principal component analysis (PCA), kinship estimation between population materials, and heatmap analysis of kinship were conducted in R. To assess population structure, ADMIXTURE software was employed with a semi-supervised learning approach that combines labeled lines with known ancestry. This information helped improve the accuracy of ancestry estimation in the unlabeled test population (Zhou et al. 2011). A neighbor-joining phylogenetic tree of the maize population was reconstructed using MEGA7 (Kumar et al. 2016) software and plotted with the R package ‘ggtree’.

### Genome-wide association study (GWAS) and detection of significant peaks

Association analysis of BLUP values was conducted using the R package ‘GAPIT’ (version 3) with the FarmCPU model (Wang and Zhang 2021). Compared to other models, such as the generalized linear model (GLM) (Yu et al. 2006) and maximum likelihood model (Zhang et al. 2010), FarmCPU integrates fixed- and random-effect models, making it particularly suitable for complex agronomic traits in maize (Liu et al. 2016). Eleven principal components and a kinship matrix were included as covariates to control for population structure and relatedness, thereby lowering the false-positive rate.

Significant SNPs were selected using *p* < 5.61 × 10^−6^ as the threshold for significant correlations, as determined through genetic type 1 error calculator (Li et al. 2012), a multiple-testing correction method designed to minimize false positives while preserving loci with substantial trait associations. To identify independent gene regions significantly associated with a target trait, the clump-based method (Crowell et al. 2016) was used; this method minimizes false peaks and detects true clusters of SNPs by filtering significantly associated SNPs, defining and clustering tightly linked SNPs. This approach mitigates the problem of multiple comparisons and avoids treating multiple, tightly linked SNPs as independent signals.

GWAS results were visualized as Manhattan plots and quantile–quantile (Q-Q) plots created with the R package ‘CMplot’ (Yin et al. 2021). Candidate genes within the upstream or downstream LD ranges of significant SNPs were identified based on their physical locations in the maize B73 reference genome (ZmB73_RefGen_v4). The appropriate LD ranges were determined based on a 50% drop in the average LD decay distance. Candidate gene predictions and functional annotation were performed using MaizeGDB (https://maizegdb.org/) and NCBI (https://www.ncbi.nlm.nih.gov/). Gene ontology (GO) analysis of candidate genes was performed on the agriGO (https://systemsbiology.cau.edu.cn/agriGOv2/index.php/manual.php) website.

### Multi-trait genome-wide association study

To evaluate the potential shared genetic architecture between agronomic traits, the cross-trait LD score regression method was employed (Bulik-Sullivan et al. 2015). This method models the expected product of the *Z*-statistics across two traits at each SNP as a linear function of the LD score as shown in the following equation:

$$\begin{array}{c}{\rm E}\left[{{Z}_{1i}Z}_{2i}|{l}_{i}\right]=\frac{\sqrt{{N}_{1}{N}_{2}{\rho }_{g}}}{M}{l}_{i}+\frac{\rho {\rm N}_{s}}{\sqrt{{N}_{1}{N}_{2}}}\end{array}(2)$$

where ${l}_{i}$ is the LD score of SNP *i*, ${N}_{1}$ and ${N}_{2}$ are the sample sizes of the two traits, ${\rho }_{g}$ is the genetic covariance between traits 1 and 2, $M$ is the number of SNPs, $\rho$ represents the phenotypic correlation due to sample overlap across studies between traits 1 and 2, and $Ns$ is the number of overlapping samples between the two GWAS datasets. ${Z}_{1i}$ and ${Z}_{2i}$ are the GWAS *Z*-statistics for traits 1 and 2 at SNP *i*, respectively, defined as:

$$\begin{array}{c}{Z}_{i}=\frac{{\beta }_{i}}{{SE}_{i}}\end{array}(3)$$

where ${\beta }_{i}$ is the estimated effect size and ${SE}_{i}$ is the standard error (SE) of SNP *i*, both derived from single-trait GWAS summary statistics. The genetic correlation ${r}_{g}$ between the traits was then estimated by normalizing the genetic covariance ${\rho }_{g}$ to the square roots of SNP-based heritabilities of the two traits. Further analysis used the MTAG tool (https://github.com/JonJala/mtag), which is based on a GLM and allows for the joint analysis of multiple traits. Covariance between traits was corrected using a variance–covariance matrix and the joint effect of multiple traits as well as the joint *p *value and effect estimate for each SNP. SNPs were filtered for significance using a genome-wide adjusted threshold of *p* < 5.61 × 10^−6^.

### Construction of the genetic network

To construct the genetic network, significant SNP peaks (named QTLs here) were identified from the GWAS results using the PLINK clumping method, with each QTL consisting of the lead SNP and its surrounding SNPs in strong LD. Inter-LD (Fang et al. 2017) was then calculated between QTL pairs using the following formula:

$$\begin{array}{c}Inter-LD=\frac{1}{2}\times \left(\frac{LD\left({QTL}_{1},{QTL}_{2}\right)}{{P}_{max}LD\left({QTL}_{1}\right)}+\frac{LD\left({QTL}_{1},{QTL}_{2}\right)}{{P}_{max}LD\left({QTL}_{2}\right)}\right)\end{array}(4)$$

in which $LD ({QTL}_{1}, {QTL}_{2})$ represents the average LD between all variants within the interval of *QTL*_1_ and those within the interval of *QTL*_2_. Additionally, ${P}_{max}LD ({QTL}_{1})$ and ${P}_{max}LD ({QTL}_{2})$ denote the maximum average LD values among any two variants within *QTL*_1_ and *QTL*_2_, respectively. LD values, calculated in PLINK (version 1.9), reflect the *r*^2^ values between specific SNPs. QTL pairs were selected with Inter-LD values of 0.4 or higher. The resulting genetic network was visualized using Cytoscape (version 3.10.3) (Shannon et al. 2003). In this network, nodes represent agronomic traits and QTLs, and edges (i.e., lines), indicate LD relationships between QTLs.

### RNA extraction and reverse-transcription quantitative PCR (RT-qPCR)

To validate the gene–gene relationships inferred from the single-trait GWAS-based genetic network, 30 gene pairs were selected that exhibited high Inter-LD (>0.8) values and were identified as high-confidence connections (edges) in the network. In total, 34 unique genes involved in 30 pairs were selected for expression tests. Total RNA was extracted from 12 tissue samples of the maize inbred line B73, collected at the flare opening (V12) and tasseling (VT) stages, between 9:00 and 10:00 a.m. Samples were ground in liquid nitrogen, and total RNA was extracted using a SteadyPure Plant RNA Extraction Kit (Accurate Biology) following the manufacturer’s instructions. RNA concentration and integrity were assessed using a spectrophotometer.

RT-qPCR analysis was performed using a SYBR Green *Pro Taq* HS Premix II Kit (Accurate Biology) with *Actin* as the internal reference transcript. Primers were designed for the 34 candidate genes (Table S7). Each reaction was carried out in a 15-μL reaction system. Amplification was conducted on an Eco Real-Time PCR System (Illumina), and gene expression levels were calculated using the ${2}^{-\Delta \Delta C\text{t}}$ method (Livak and Schmittgen 2001).

## Supplementary Information

Below is the link to the electronic supplementary material.

Supplementary file1 (DOCX 1348 KB)

Supplementary file2 (XLSX 343 KB)

## Declarations

### Data availability

The data that support the findings of this study are available from the corresponding author upon reasonable request.
