# Supplementary material for: Dissecting the genetic basis of agronomic traits by multi-trait GWAS and genetic networks in maize (Zea mays L.)
Source: aBIOTECH. 2025 Aug 14;6(4):707–25. doi: 10.1007/s42994-025-00241-4 (PMC12647461; doi:10.1007/s42994-025-00241-4)
Supplement: Supplementary file 1 — Supplementary file1 (DOCX 1348 KB) [file 42994_2025_241_MOESM1_ESM.docx]

**Dissecting the genetic basis of agronomic traits by multi-trait GWAS and genetic networks in maize (*Zea mays* L.)**

Ying Zhou^1,2,3^ ^†^, Yanfang Heng^2^ ^†^, Shoukun Chen^2,3^, Jinglu Wang^4^, Kunhui He^2,3^, Jiahui Geng^2,3^, Kaijian Fan^2^, Yonggui Xiao^2^, Changling Huang^2,3^, Jiankang Wang^2,3^, Enying Zhang^1,*^, Liang Li^2,*^, Huihui Li^2,3,*^

^1^ College of Agronomy, Qingdao Agricultural University, Qingdao 266109, China

^2^ State Key Laboratory of Crop Gene Resources and Breeding, Institute of Crop Sciences, Chinese Academy of Agricultural Sciences (CAAS), CIMMYT-China office, Beijing 100081, China

^3^ Nanfan Research Institute, CAAS, Sanya, Hainan 572024, China.

^4^ Beijing Key Lab of Digital Plant, Information Technology Research Center, Beijing Academy of Agriculture and Forestry Sciences, Beijing 100097, China

^†^These authors contributed equally to this study.

*Correspondences: Huihui Li (lihuihui@caas.cn); Liang Li ([liliang05@caas.cn](mailto:liliang05@caas.cn)); Enying Zhang (eyzhang@qau.edu.cn)


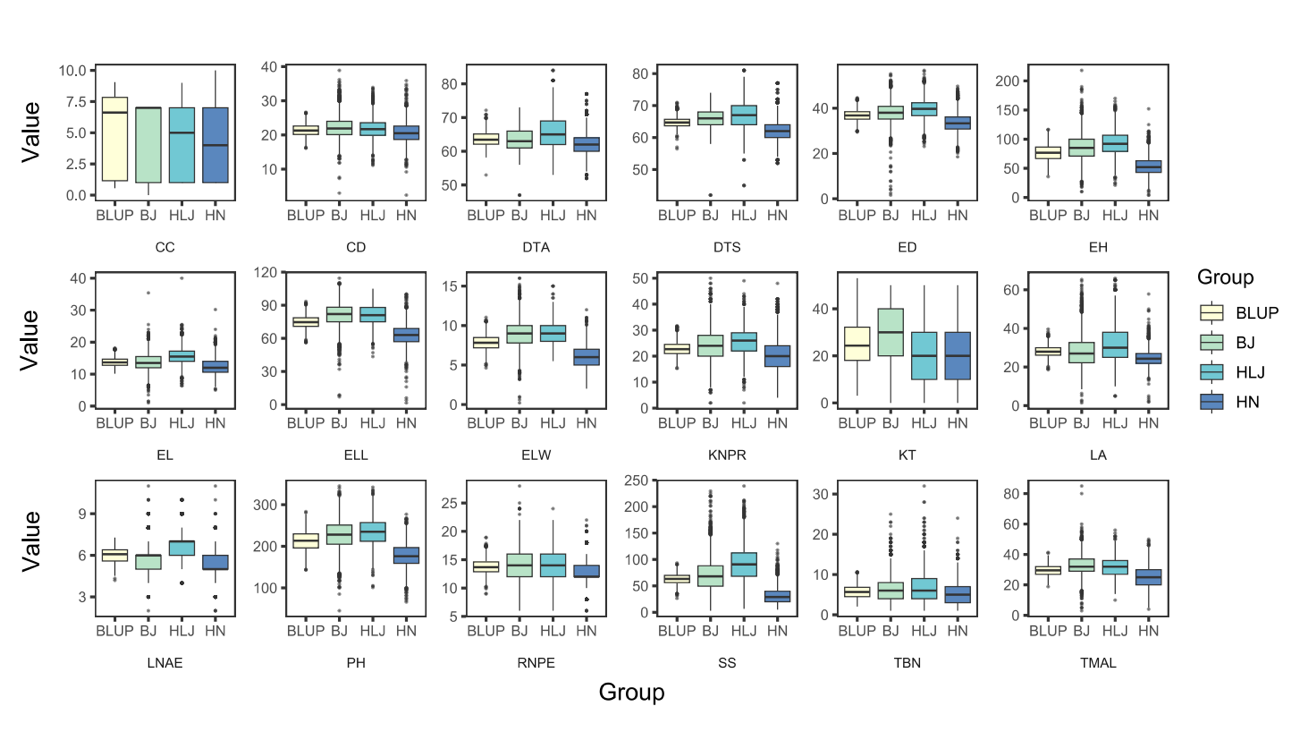


**Fig. S1** Boxplots of phenotypic values measured at the BJ, HN, and HLJ experimental sites and BLUP values for each of the 18 maize agronomic traits.

Boxplots showing the median, interquartile range, and outliers for each trait. **BJ**, Beijing; HN, Henan; **HLJ**, Heilongjiang; **BLUP**, best linear unbiased prediction; **CC**, cob color; **CD**, cob diameter; **DTA**, days to anthesis; **DTS**, days to silking; **ED**, ear diameter; **EL**, ear length; **ELL**, ear leaf length; **ELW**, ear leaf width; **EH**, ear height; **KNPR**, kernel number per row; **KT**, kernel type; **LA**, leaf angle; **LNAE**, leaf number above ear; **PH**, plant height; **RNPE**, row number per ear; **SS**, stalk strength; **TBN**, tassel branch number; **TMAL**, tassel main axis length.

**
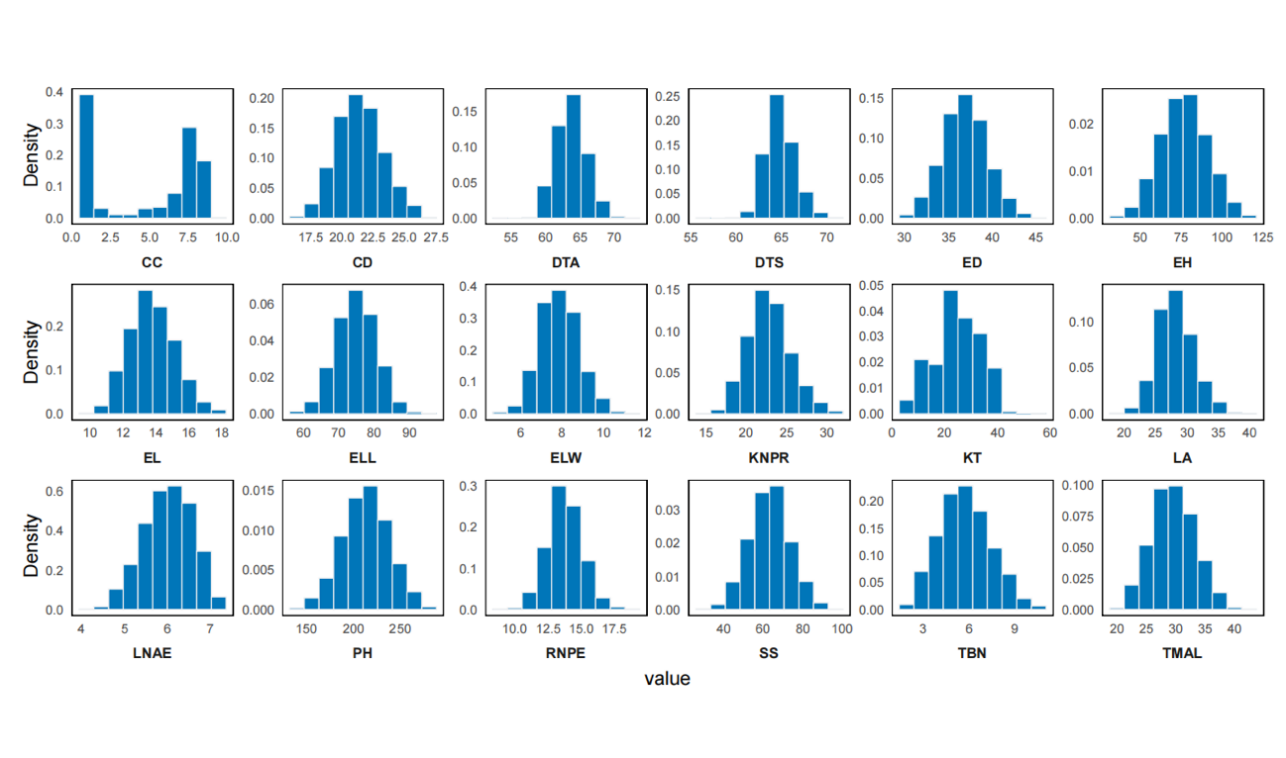
**

**Fig. S2** Data distribution histogram of phenotypic values for each of the 18 maize traits.

**CC**, cob color; **CD**, cob diameter; **DTA**, days to anthesis; **DTS**, days to silking; **ED**, ear diameter; **EL**, ear length; **ELL**, ear leaf length; **ELW**, ear leaf width; **EH**, ear height; **KNPR**, kernel number per row; **KT**, kernel type; **LA**, leaf angle; **LNAE**, leaf number above ear; **PH**, plant height; **RNPE**, row number per ear; **SS**, stalk strength; **TBN**, tassel branch number; **TMAL**, tassel main axis length.

**
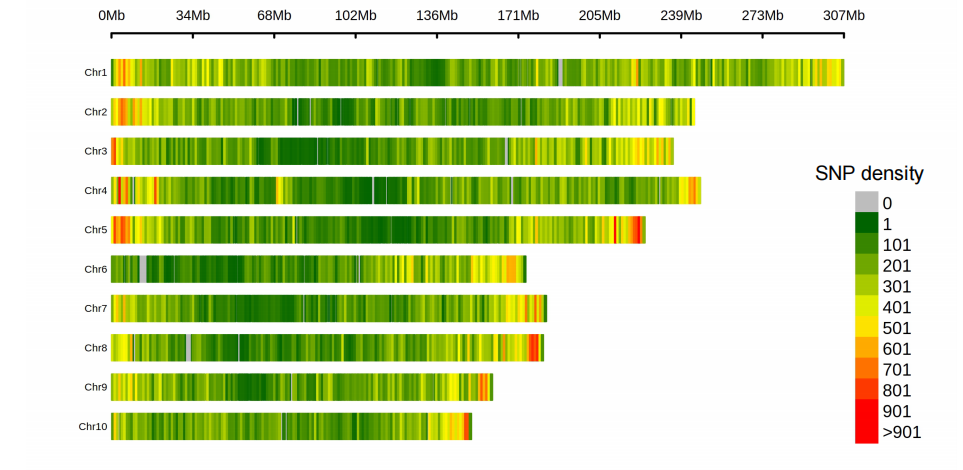
Fig. S3** Density distribution of SNPs on each maize chromosome.

**
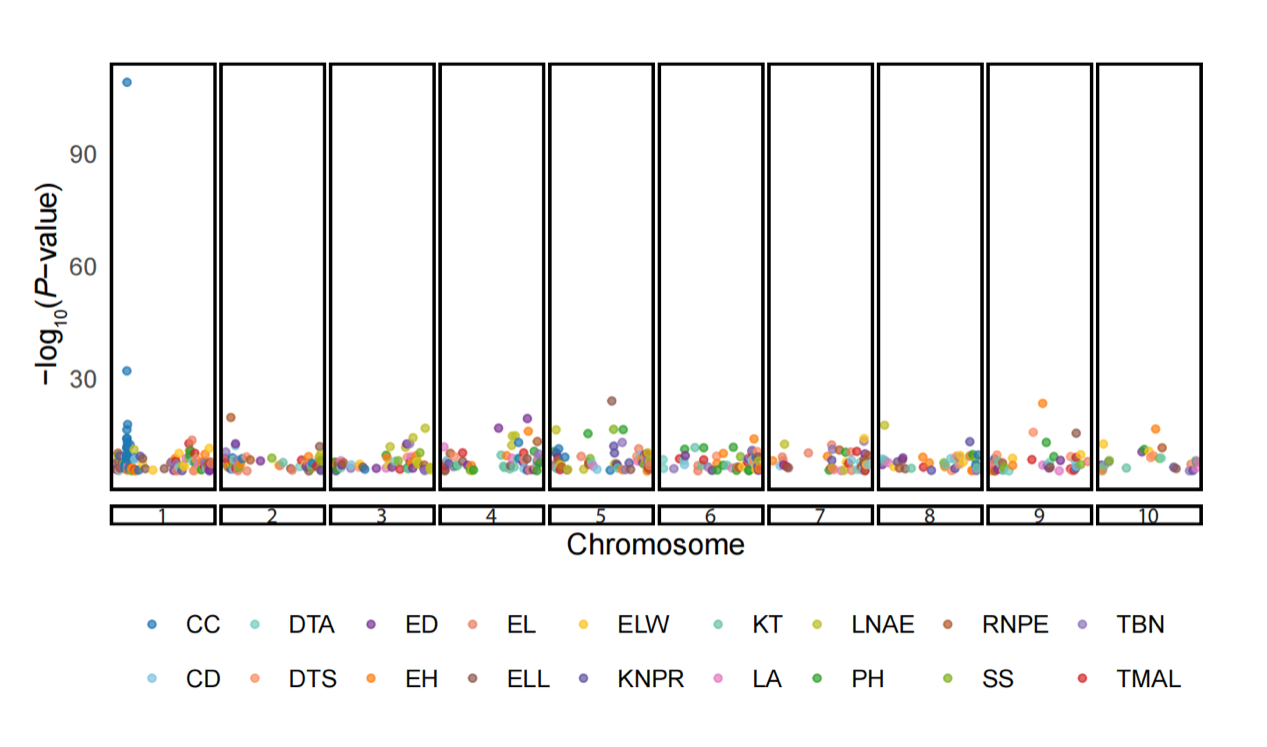
**

**Fig. S4** Manhattan plot showing significant SNPs (*p* < 5.61 × 10⁻^6^) identified by the FarmCPU model across 18 maize traits.

Each point represents a significant SNP, with the –log_10_(*P*-value) on the y-axis and its physical position on the x-axis. Different colors indicate different traits. Chromosomes are ordered sequentially along the x-axis. **CC**, cob color; **CD**, cob diameter; **DTA**, days to anthesis; **DTS**, days to silking; **ED**, ear diameter; **EL**, ear length; **ELL**, ear leaf length; **ELW**, ear leaf width; **EH**, ear height; **KNPR**, kernel number per row; **KT**, kernel type; **LA**, leaf angle; **LNAE**, leaf number above ear; **PH**, plant height; **RNPE**, row number per ear; **SS**, stalk strength; **TBN**, tassel branch number; **TMAL**, tassel main axis length.

**
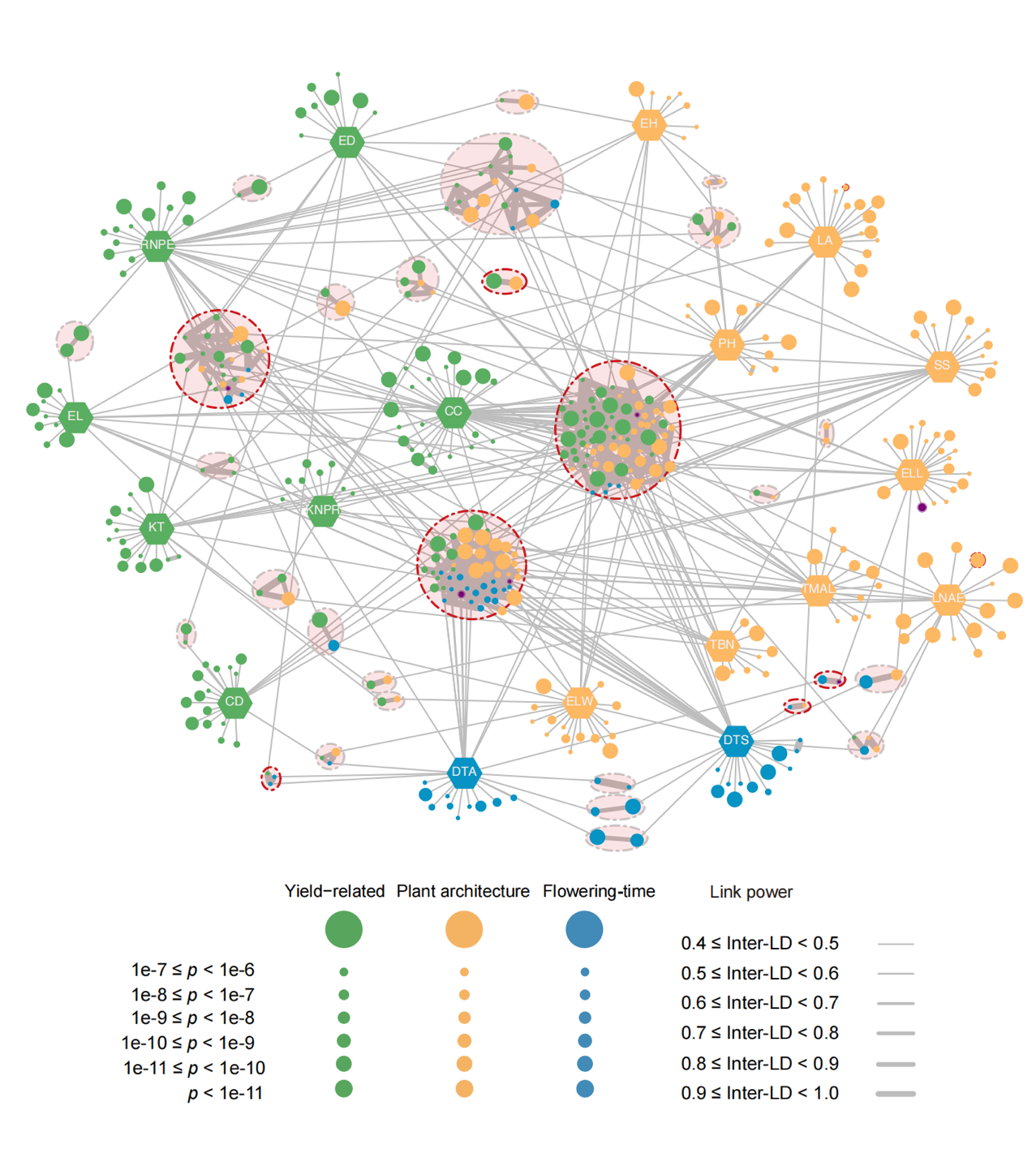
**

**Fig. S5** Genetic networks across different maize traits based on loci detected by MTAG.

Solid hexagons, traits; filled circles, QTLs; hollow ellipses, hubs; red dashed boxes, QTL intervals covering known genes. The nodes represent 18 traits and their responsible QTLs. Edges between QTLs from different traits are linked by Inter-LD. Only edges with an average LD ≥ 0.4 are displayed. **CC**, cob color; **CD**, cob diameter; **DTA**, days to anthesis; **DTS**, days to silking; **ED**, ear diameter; **EL**, ear length; **ELL**, ear leaf length; **ELW**, ear leaf width; **EH**, ear height; **KNPR**, kernel number per row; **KT**, kernel type; **LA**, leaf angle; **LNAE**, leaf number above ear; **PH**, plant height; **RNPE**, row number per ear; **SS**, stalk strength; **TBN**, tassel branch number; **TMAL**, tassel main axis length.
